# Effect of cue validity on the contextual cueing effect

**DOI:** 10.3389/fpsyg.2024.1495780

**Published:** 2024-12-10

**Authors:** Wen Su, Guang Zhao, Jie Ma

**Affiliations:** ^1^Public Courses Teaching Department, Guangzhou Sport University, Guangzhou, China; ^2^Faculty of Psychology, Tianjin Normal University, Tianjin, China; ^3^College of Education Science, Hubei Normal University, Huangshi, China

**Keywords:** visual search, context, contextual cueing effect, cue validity, learning

## Abstract

**Purpose:**

In daily life, people are adept at extracting task-relevant information from complex visual environment to guide attention more effectively toward the target. This process underpins the contextual cueing effect, where repeated exposure allows individuals to learn associations between contextual cues and targets, thereby enhancing visual search efficiency. However, the cue validity of context —how consistently cues predict target locations—is not always guaranteed in real life. This study focused on cue validity as a critical factor in understanding the contextual cueing effect. Within the study of contextual cueing, cue validity specifically refers to the probability that contextual cues accurately indicate the location of a target.

**Methods:**

In Experiment 1, we manipulated three levels of cue validity (100, 75, and 50%) using a classic contextual cueing paradigm. Experiment 2 examined the potential impact of an imbalanced predictable *vs* unpredictable trial ratio. In Experiment 3, we explored whether the absence of the contextual cueing effect was due to unsuccessful learning or unsuccessful later expression.

**Results:**

Results from Experiment 1 revealed that higher cue validity (100 and 75%) significantly elicited the contextual cueing effect, resulting in faster responses for repeated displays, whereas lower cue validity (50%) did not result in this effect because the repeated displays could not be effectively learned. Experiment 2 showed that the contextual cueing effect remained robust despite an imbalanced ratio of predictable to unpredictable displays. Experiment 3 further showed that low cue validity affects the early learning phase of context-target associations rather than the later expression in visual search.

**Conclusion:**

Our study highlights the significant role of cue validity in implicit learning from visual cues. High cue validity enhances learning by providing highly stable context-target associations, while low cue validity does not actively facilitate attention allocation, thereby not promoting the learning process. These findings underscore the importance of cue validity in processing visual information.

## 1 Introduction

In our daily lives, we are surrounded by a wealth of visual information that integrates with surrounding elements to form a “scene” or “context.” Previous research has highlighted the facilitative role of context in visual search (Biederman et al., 1982). Individuals can locate a target (e.g., a car) more rapidly in a context where the target is more likely to be found (e.g., a street) compared to a less likely context (e.g., a kitchen). Beyond object features, spatial cues provided by context can also significantly enhance the efficiency of visual search. Repeated exposure to specific spatial arrangements of targets and distractors in such tasks could lead to quicker responses—a phenomenon known as “contextual cueing effect” (Chun and Jiang, 1998). For example, after repeated visits, quickly finding your favorite fruit, apples, in a grocery store becomes easy due to the consistent aisle organization in the store. This illustrates the contextual cueing effect, where familiar spatial arrangement speeds up visual searches through learned cues.

The contextual cueing effect is a robust and long-lasting visual learning mechanism, persisting over weeks (Chun and Jiang, 2003). Remarkably, such learning can occur after only a few repetitions during a visual search (Brockmole et al., 2006; Geyer et al., 2010; Xie et al., 2020; Seitz et al., 2023). Meanwhile, it appears to be independent of individual differences in intelligence (Merrill et al., 2014) and remains intact in clinical populations with psychological and neurological disorders (Lamy et al., 2008; Jiménez-Fernández et al., 2011; Oudman et al., 2011; Kourkoulou et al., 2012; Jiang et al., 2019). Moreover, the effect has been observed across species, including baboons (Goujon and Fagot, 2013) and pigeons (Wasserman et al., 2014), making it a powerful tool for investigating implicit statistical learning mechanisms (Goujon et al., 2015). Various factors have been found to modulate the size of the contextual cueing effect (Jiang and Sisk, 2019), such as the properties of the local context around the target (Olson and Chun, 2002; Brady and Chun, 2007), the number of targets (Kunar and Wolfe, 2011; Zellin et al., 2011), the signal-to-noise ratio (Yang and Merrill, 2015), the spatial constraints (Olson and Chun, 2002; Brady and Chun, 2007; Couperus et al., 2011), working memory load (Annac et al., 2013; Pollmann, 2019), and selective attention (Jiang and Leung, 2005).

Studies highlighted the critical role of selective attention in the implicit learning of visual cues and the contextual cueing effect (Jiang and Leung, 2005; Couperus et al., 2011; Sisk et al., 2019). Selective attention enhances focus on the task-relevant information while filtering out irrelevant distractions. Through repeated exposure, individuals can learn stable spatial relationships between context cues and targets, allowing them to direct attention efficiently to target locations (Olson and Chun, 2002). Importantly, varying the proportion of useful cues and noises within a single visual display trial does not affect the generation of contextual cueing effect (Couperus et al., 2011; Yang and Merrill, 2015). Even when only a fraction of the cues in the whole context is predictive of target location, individuals effectively extract this information and exhibit a strong contextual cueing effect (Olson and Chun, 2002; Brady and Chun, 2007). These findings collectively indicate that selective attention facilitates the extraction of consistently valid information from noisy or distracting visual contexts, thus promotes behavioral responses.

Previous studies on visual context have predominantly focused on fully valid scenarios where the spatial relationship between cues and targets are fixed and consistent (Jiang and Sisk, 2019). This approach has provided valuable insights into how predictable environments enhance visual searching performance. However, real-world cues are rarely fully predictable. Nevertheless, the human brain demonstrates a remarkable ability to effectively utilize those incompletely predictable cues to guide behavioral responses. Cue validity, which refers to the probability of cues predicting the location of a target, has been found to be a crucial factor influencing how effectively cues guide attention to target locations (Small et al., 2003; Vossel et al., 2006; Jamet, 2014; Lou et al., 2022). Extensive research has shown that valid cues significantly enhance attention performance than invalid cues, typically reflected by faster reaction times (RTs) and more efficient search performance (Posner et al., 1980; Posner and Petersen, 1990; Vossel et al., 2006). High cue validity improves attention allocation by allowing individuals to focus cognitive resources more effectively on the likely location of the target (Luck et al., 1996; Girardi et al., 2013).

Despite the well-recognized effects of the cue validity on attention allocation (Luck et al., 1996; Girardi et al., 2013), its impact on the contextual cueing effect remains uncertain. Previous studies have examined that varying the proportion of useful cues and noise within a single trial does not significantly impact contextual cueing (Olson and Chun, 2002; Brady and Chun, 2007; Couperus et al., 2011; Yang and Merrill, 2015). However, it is still unclear whether the cue validity across multiple display trials would influence the contextual cueing effect. Our study aimed to examine this by manipulating cue validity across various trials. In Experiment 1, we examined the impact of different cue validity levels (100, 75, and 50%) on the contextual cueing effect. Experiment 2 was built on Experiment 1 and investigated whether the lack of contextual cueing effect in the 50% cue validity condition was due to low cue validity or an imbalance between predictable and unpredictable trials. Thus, Experiment 2 adjusted the trial ratio to 1:3 with 100% cue validity to explore this effect. In Experiment 3, we further explored the role of cue validity by separating the learning and expression phases to understand its impact at different stages. We hypothesize that lower cue validity reduces attentional focus on potential target location and impairs the acquisition of visual context cues, resulting in minimal or no contextual cueing effect. Our research aims to explore how varying cue validity affects the ability to learn and utilize contextual cues, shedding light on the adaptability of visual attention and learning processes in dynamic environments.

## 2 Experiment 1

Experiment 1 aimed to explore how different cue validity affects the contextual cueing effect. We employed the classic contextual cueing paradigm (Chun and Jiang, 1998; Vadillo et al., 2016; Jiang and Sisk, 2019) and established three sub-experiments to explore different levels of cue validity: 100, 75, and 50%.

### 2.1 Methods

#### 2.1.1 Participants

A total of 74 healthy participants were recruited and randomly assigned to three sub-experiments, each with a different level of cue validity: Experiment 1a with 100% cue validity (*n* = 25; 4 males and 21 females; ages 18–26 years; mean age ± SD = 20.20 ± 2.12 years), Experiment 1b with 75% cue validity (*n* = 24; 4 males and 20 females; ages 18–27 years; mean age ± SD = 20.46 ± 2.23 years), and Experiment 1c with 50% cue validity (*n* = 25; 5 males and 20 females; ages 18–30 years; mean age ± SD = 21.32 ± 3.09 years). The three groups did not differ in terms of sex and age. All participants were right-handed, with normal or corrected vision, and had no prior experience in similar experiments. They were unaware of the study’s purpose and received compensation after study participation. Previous studies observed the contextual cueing effect with a large effect size, with Cohen’s *d* ranging from 0.84 to 0.99 (Colagiuri and Livesey, 2016). A G*Power analysis (Erdfelder et al., 1996) confirmed that the sample size for each sub-experiment was appropriate, achieving a power of 0.8 with 14 participants and 0.95 with 21 participants. This study was approved by the Ethics Committee of the School of Psychology, Liaoning Normal University.

#### 2.1.2 Apparatus and materials

The experiment was programmed using PsychoPy (Peirce, 2007, 2009), a Python library for designing psychological experiments. Stimuli were displayed on a 19-inch monitor with a resolution of 1,024 × 768 pixels and a refresh rate of 60 Hz. Participants were seated about 60 cm away from the screen.

**Figure 1:**
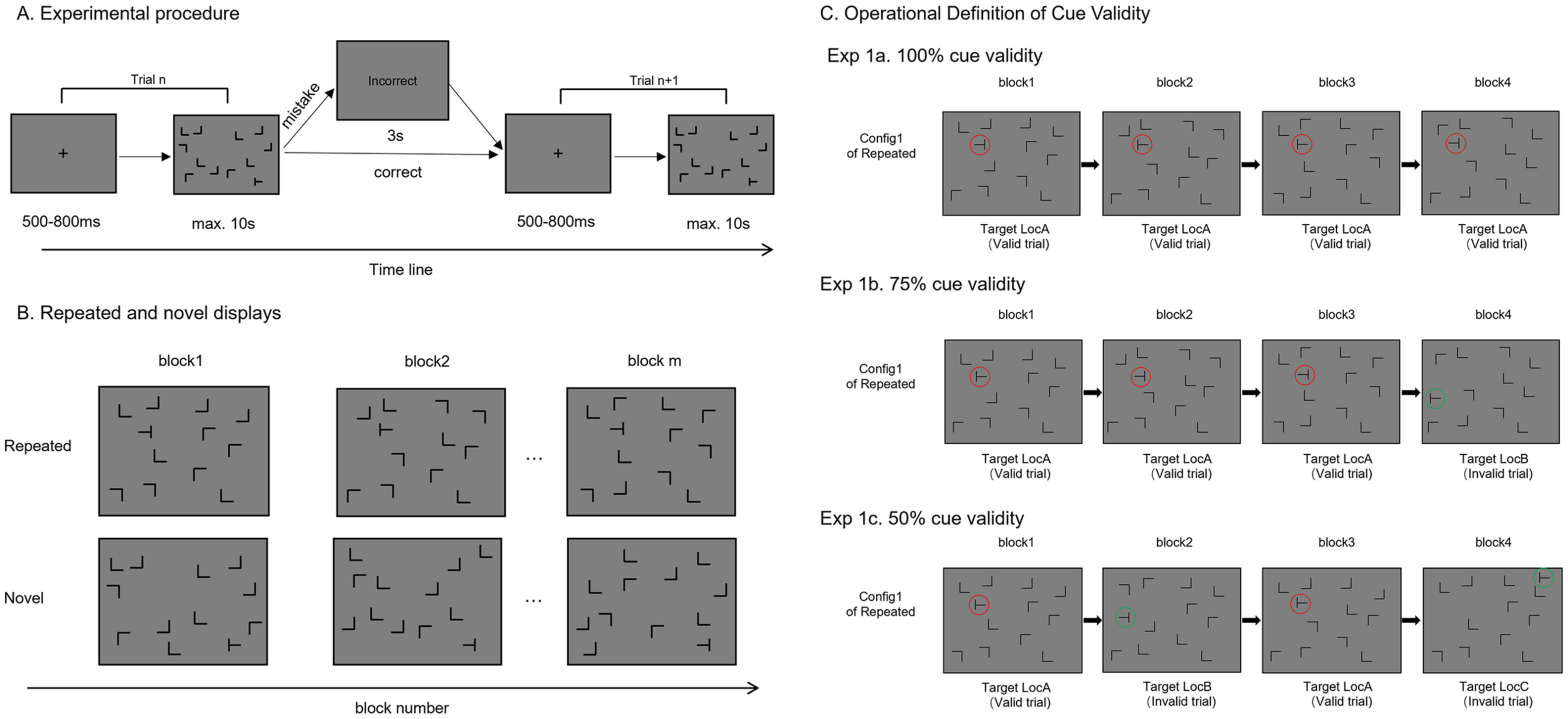
Schematic illustration of experimental procedure and context conditions. **(A)** The trial begins with a central cross displayed for 500–800 ms and followed by a 10-s search display. Participants are instructed to identify the orientation of target “T” among rotating “L” distractors. Correct responses lead directly to the next trial, while incorrect responses lead to a 3-s feedback delay. **(B)** In the repeated context condition, distractor locations are constant in the same configuration, with varying orientations. Conversely, novel context condition has random distractor locations and orientations, but a consistent target location. **(C)** Operational definition of cue validity. The upper panel presents the experimental setup in Experiment 1a, showing the spatial distribution of a repeated display over 4 blocks under 100% cue validity condition. Here, a repeated configuration (Config 1) consistently matches a fixed target location (Loc A) with 100% probability in an epoch. The middle panel depicts the experimental setup in Experiment 1b, demonstrating the spatial distribution of a repeated display over 4 blocks under 75% cue validity condition. In this scenario, a repeated configuration (Config 1) has a 75% probability of matching a fixed target location (Loc A) and a 25% probability of appearing at random locations (Loc B). The lower panel illustrates the experimental setup in Experiment 1c, showing the spatial distribution of a repeated context over 4 blocks under 50% cue validity condition. This indicates that a repeated configuration (Config 1) has a 50% probability of matching a fixed target location (Loc A) and a 50% probability of appearing at other random locations (Loc B and C). Red circles mark the fixed target location, while green circles signify the random target locations.

The search display consisted of 12 black items on a grey background: one “T”-shaped target and eleven “L”-shaped distractors, each measuring 2.14° × 2.14°. The horizontal and vertical lines of the ‘L’ distractors were matched in length. The target’s orientation was randomized by rotating the ‘T’ letter either 90° or 270° from the vertical midline, and the ‘L’ distractors were randomly rotated by 0°, 90°, 180°, or 270° in each trial (Figure 1). All items were arranged in an 8 × 10 invisible grid (23.81° × 18.04°), evenly distributed across four quadrants to avoid positional bias. To prevent explicit collinearity effects, items were slightly jittered vertically or horizontally by ±0.11° (Jiang et al., 2000; Kawahara, 2007).

#### 2.1.3 Design and procedure

Each trial began with a central fixation cross that presented for 500–800 ms, followed by a 10-s search display (Figure 1A). Participants were instructed to identify the target “T” among “L” distractors and respond to its orientation using the “F” and “J” keys. The mapping between target orientation and response keys was counterbalanced across participants. Participants were required to respond as quickly and accurately as possible. Correct responses led directly to the next trial, while incorrect responses resulted in a 3-s feedback delay.

Three independent sub-experiments were conducted, each corresponding to a different level of cue validity (100, 75, 50%). In each sub-experiment, context condition (repeated, novel) served as the within-subjects factor. In the repeated displays, distractors consistently appeared in the same locations, though their orientations could vary (Figure 1B). This approach is consistent with the methods used by Zhao et al. (2021), aiming to minimize reliance on distractor attributes through randomizing orientations and emphasize the learning of spatial configurations. There were 16 distinct configurations for the repeated displays, each maintaining a fixed pattern of distractor locations. In contrast, the novel displays presented randomly positioned distractors with varying orientations, but a consistent target location (Figure 1B). Each cue validity condition consisted of 896 trials, divided into 28 blocks. Each block contained 16 repeated and 16 novel trials, presented in a random order. Each configuration of the repeated display appeared once per block, resulting in a total of 28 presentations across blocks.

To ensure that the target appeared equally in all four quadrants, eight locations from each quadrant were chosen randomly—four for the repeated conditions and four for the novel conditions—resulting in a total of 32 locations. The target locations in the repeated and novel displays were distinct from each other. Repeated displays consisted of 16 unique configurations, each corresponds to a specific target location (when valid, as further explained below regarding cue validity settings). Similarly, in novel displays, despite the changes in the locations and orientations of the distractors, 16 fixed target locations were used, with each location appearing once per block and in total 28 times across blocks. To enhance statistical power, 28 blocks were grouped into 7 epochs (4 blocks per epoch) for data analysis.

Cue validity levels were defined as the probability of repeated displays predicted target location in an epoch (four blocks). In Experiment 1a, with 100% cue validity, a configuration of repeated display always matched a fixed target location, aligning with the classical paradigm (Figure 1C, upper panel). In Experiment 1b, with 75% cue validity, a configuration of repeated display matched a fixed target location in three out of four blocks, while in the remaining block, the target appeared at a randomly selected location, giving a 75% probability of accurate prediction (Figure 1C, middle panel). The unexpected locations were chosen from the 48 unused locations of the 80-location search display, excluding the 32 fixed target locations. Similarly, in Experiment 1c, with 50% cue validity, the target location was consistent in only half of the trials of the repeated displays, with the other half randomly assigned to unexpected locations (Figure 1C, lower panel). Thus, each repeated display had an equal probability of being valid (predicting the fixed target location) or invalid (random target location). In the 75 and 50% cue validity conditions, the presentation order of valid (predictable) and invalid (unpredictable) repeated displays was randomized within each epoch, across four blocks.

### 2.2 Results

**Figure 2:**
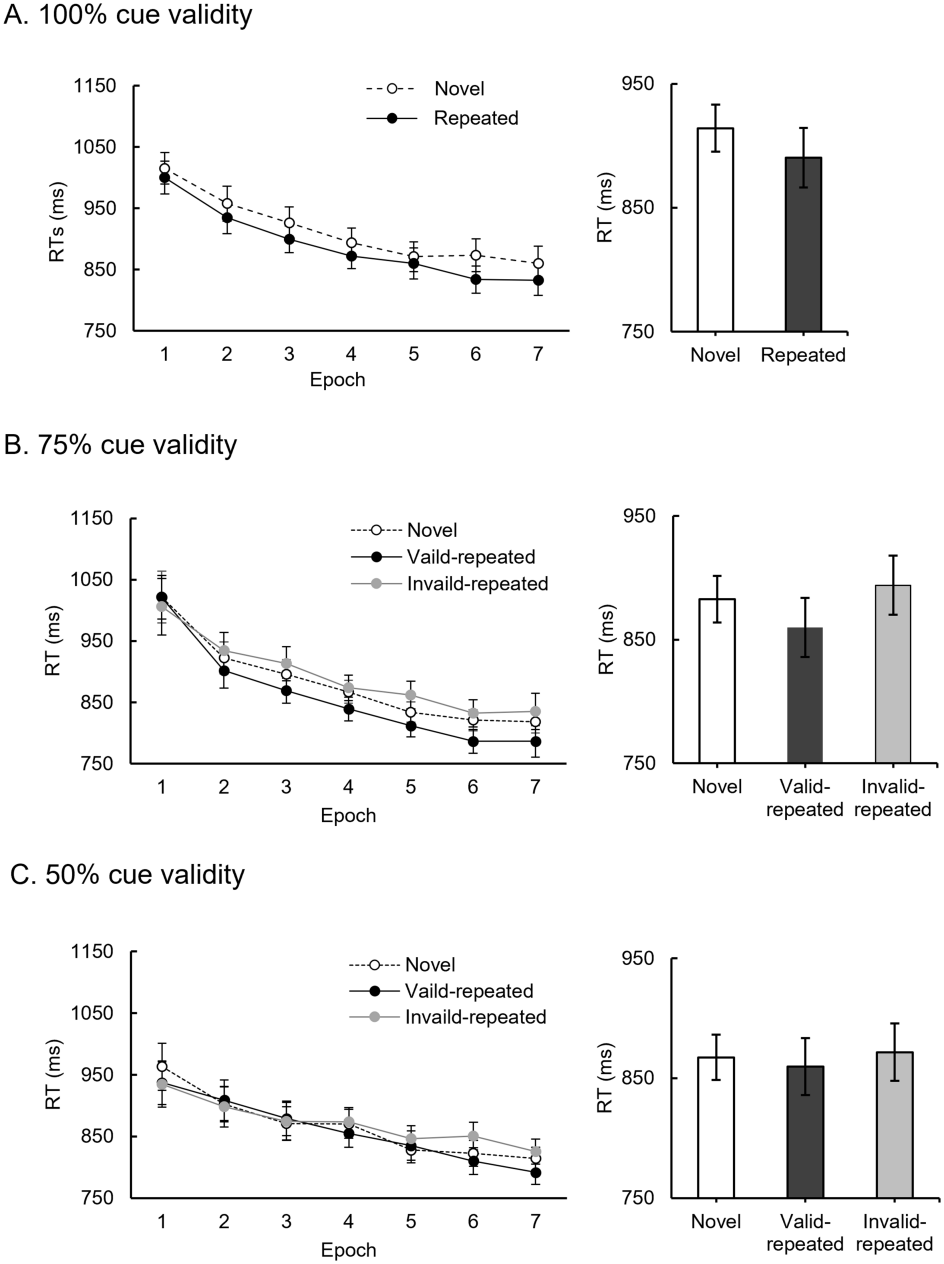
Mean RTs for each cue validity condition in Experiment 1. **(A)** Results for Experiment 1a under the 100% cue validity condition, including novel and repeated displays. The dashed line represents novel displays, and the solid line represents repeated displays. **(B)** Results for Experiment 1b under the 75% cue validity condition, including novel, valid repeated, and invalid repeated displays. The dashed line is for novel displays, and the solid line is for repeated displays, and the gray line represents the invalid repeated displays. **(C)** Results for Experiment 1c under the 50% cue validity condition, including novel, valid repeated, and invalid repeated displays. The dashed line is for novel displays, and the solid line is for repeated displays, and the gray line represents the invalid repeated display. Mean RTs and standard errors are shown as a function of epoch and context condition on the left panel. The overall mean RTs for each context condition are shown on the right panel, encompassing all epochs.

Data preprocessing involved excluding omission, incorrect responses, and outlier trials with RTs outside the mean RT ± 3 SDs or below 200 ms. This led to the exclusion of 3% of trials in Experiment 1a, 3.1% of trials in Experiment 1b, and 3.1% of trials in Experiment 1c. Figure 2 showed the mean RTs across epochs and context conditions for each cue validity group. Specifically, the mean RTs represent the average RTs calculated for each participant within each condition and epoch, subsequently averaged across all participants to derive the presented results.

#### 2.2.1 Experiment 1a: 100% cue validity

A 2 (context condition: repeated and novel) × 7 (epoch) repeated-measures analysis of variance (ANOVA) was performed, with a Greenhous-Geisser correction where necessary. The results for the 100% cue validity (Figure 2A) revealed a significant main effect of context condition, *F* (1, 24) = 5.82, *p* < 0.05, *η_p_^2^* = 0.20, *BF_10_* = 2.63, indicating a contextual cueing effect with faster RTs for repeated displays (mean RT ± SD: 890.36 ± 106.21 ms) compared to novel displays (913.93 ± 117.86 ms). The main effect of epoch was also significant, *F* (3.01, 72.27) = 34.89, *p* < 0.001, *η_p_^2^* = 0.59, *BF_10_* = 5.43 × 10^22^, indicating a practice effect, as participants responded faster over time during the experiment. However, the interaction between context condition and epoch was not significant, *F* (3.86, 92.58) = 0.84, *p* = 0.50, *η_p_^2^* = 0.03, *BF_10_* = 0.05. To address concerns about potential configuration benefits between novel and repeated trials, we calculated the difference between these trials in the first and second blocks. The results for the first block showed no significant difference between novel and repeated trials, *t* (24) = 0.97, *p* = 0.34, Cohen’s *d* = 0.19, *BF_10_* = 0.32. Similarly, the results for the second block also indicated no significant difference, *t* (24) = −0.19, *p* = 0.85, Cohen’s *d* = −0.04, *BF_10_* = 0.21. Therefore, we conclude that the non-significant interaction is not due to potential configuration benefits, but rather reflects robust learning effect. It is noteworthy that repeated displays typically provide a behavioral advantage of 50–100 ms compared to novel displays (Chun and Jiang, 1998; Jiang and Sisk, 2019). However, our results showed a smaller behavioral advantage of approximately 24 ms. This is likely due to our adaptation of the experimental design from Zhao et al. (2021), where the orientation of distractors in repeated displays was randomized to emphasize the learning of spatial configurations. Consequently, minimizing the influence of distractor attributes led to a reduction in the observed behavioral advantage.

#### 2.2.2 Experiment 1b: 75% cue validity

Similar to Experiment 1a, a 2 (context condition: repeated and novel) × 7 (epoch) repeated-measures ANOVA was conducted. For the 75% cue validity, only the main effect of epoch was significant, *F* (1.71, 39.29) = 32.04, *p* < 0.001, *η_p_^2^* = 0.58, *BF_10_* = 5.86 × 10^20^. There was no significant effect of context condition, *F* (1, 23) = 2.61, *p* = 0.12, *η_p_^2^* = 0.10, *BF_10_* = 0.78, nor a significant interaction, *F* (6, 138) = 0.42, *p* = 0.86, *η_p_^2^* = 0.02, *BF_10_* = 0.02.

However, the above results do not necessarily suggest that participants could not learn context cues under the 75% cue validity. The absence of a contextual cueing effect may have been due to interference from invalid repeated displays, where random target locations disrupted the spatial relationship between target and distractors. Consequently, attention might not been effectively directed to unexpected target locations in invalid trials, leading to RTs comparable to or slower than those for novel displays. The intermixing of valid and invalid repeated displays could therefore obscure the benefits conferred by the valid repeated contexts. To assess this hypothesis, we conducted further analyses which separated RTs for valid and invalid repeated displays.

A 3 (context condition: novel, valid repeated, invalid repeated displays) × 7 (epoch) repeated-measures ANOVA was performed (Figure 2B). A significant main effect of context condition was found, *F* (2, 46) = 6.29, *p* < 0.005, *η_p_^2^* = 0.22, *BF_10_* = 7.58, with marginally significantly faster RTs for valid repeated displays (859.54 ± 98.25 ms) compared to novel (882.97 ± 99.79 ms, mean difference = −23.43 ms, SE = 10.49, 95% CI = −50.5 ms to 3.65 ms, *p* = 0.07, Cohen’s *d* = −0.19) and invalid repeated displays (893.83 ± 105.07 ms, mean difference = −34.29 ms, SE = 9.91, 95% CI = −59.86 ms to −8.71 ms, *p* = 0.006, Cohen’s *d* = 0.27). The difference in RTs between the invalid repeated displays (893.83 ± 105.07 ms) and novel displays (882.97 ± 99.79 ms) was not significant, mean difference = 10.86 ms, SE = 9.20, 95% CI = −12.89 ms to 34.61 ms, *p* = 0.25, Cohen’s *d* = 0.09. The Holm-Bonferroni correction was used to adjust for multiple testing. The main effect of epoch was also significant, *F* (1.95, 44.83) = 26.47, *p* < 0.001, *η_p_^2^* = 0.54, *BF_10_* = 7.18 × 10^17^. Similar to what found in the 100% cue validity condition, there was no significant interaction between context condition and epoch, *F* (4.95, 113.75) = 1.14, *p* = 0.34, *η_p_^2^* = 0.05, *BF_10_* = 0.03. These results indicate a contextual cueing effect when cues had a 75% probability of predicting the target location. Participants successfully learned the association between repeated configurations and fixed target locations under the 75% cue validity condition.

#### 2.2.3 Experiment 1c: 50% cue validity

As in previous sub-experiments, a 2 (context condition: repeated and novel) × 7 (epoch) repeated-measures ANOVA was used. Only the main effect of epoch was significant, *F* (2.79, 66.99) = 20.69, *p* < 0.001, *η_p_^2^* = 0.46, *BF_10_* = 2.91 × 10^14^, while no significant effect of context condition, *F* (1, 24) = 0.04, *p* = 0.84, *η_p_^2^* = 0.002, *BF_10_* = 0.23, or the interaction, *F* (3.43, 82.24) = 1.59, *p* = 0.19, *η_p_^2^* = 0.06, *BF_10_* = 0.21.

To avoid the interference from invalid repeated displays, a 3 (context condition: novel, valid repeated, invalid repeated displays) × 7 (epoch) repeated-measures ANOVA was performed (Figure 2C). The significant main effect of epoch was observed, *F* (3.08, 73.87) = 18.94, *p* < 0.001, *η_p_^2^* = 0.44, *BF_10_* = 2.06 × 10^13^, and a significant interaction between context condition and epoch was found, *F* (12, 288) = 1.97, *p* < 0.05, *η_p_^2^* = 0.08, *BF_10_* = 0.62. Further tests showed that neither invalid repeated displays nor valid repeated displays demonstrated a reaction advantage compared to novel displays in any epoch (*ps* > 0.05, Holm-Bonferroni corrected). A Bayes factor of 0.62 also indicates moderate evidence in favor of the null hypothesis. In addition, the main effect of context condition was not significant, *F* (2.48) = 0.84, *p* = 0.44, *η_p_^2^* = 0.03, *BF_10_* = 0.14. These results suggest no contextual cueing effect under 50% cue validity.

Overall, the 50% cue validity group did not show a contextual cueing effect, whether repeated configurations matched fixed or random target locations. In contrast, contextual cueing effects were observed in the 100 and 75% groups when paired with fixed target locations.

## 3 Experiment 2

Experiment 1 demonstrated that high cue validity conditions (100 and 75%) significantly elicited the contextual cueing effect, whereas this effect was not observed in 50% cue validity. Previous research indicates that the ratio of validly to invalidly cued targets influences attentional allocation (Vossel et al., 2006), and the proportion of repeated and novel displays modulates the magnitude of the contextual cueing effect (Yang and Merrill, 2015). This implies that an imbalance in the number of predictable and unpredictable trials may prevent participants from learning contextual cues. Specifically, if participants consider invalid repeated displays as unpredictable, as similar to novel trials, the ratio of predictable (valid repeated) to unpredictable (both invalid repeated and novel) becomes 1:3 under the 50% cue validity condition. This disproportion in the number of predictable and unpredictable may contribute to the absence of the contextual cueing effect. Therefore, it remains unclear whether the lack of the contextual cueing effect in 50% cue validity group was due to low cue validity or if the imbalance between predictable displays to unpredictable displays hampered the extraction of valid cues. To investigate this question, Experiment 2 adjusted the number of repeated and novel trials to a 1:3 ratio, ensuring that repeated displays had 100% cue validity. This setup aimed to examine the impact of display imbalance on the contextual cueing effect when valid cues are less frequent.

### 3.1 Methods

#### 3.1.1 Participants

Twenty-four healthy participants were recruited (4 males and 20 females; ages 18–23 years; mean age ± SD = 19.79 ± 1.35 years). A G*Power analysis confirmed the sample size was appropriate, achieving a power of 0.80 with 14 participants and 0.95 with 21 participants. All other information regarding the participants is same as in Experiment 1.

#### 3.1.2 Apparatus and materials

Same as Experiment 1.

#### 3.1.3 Design and procedure

In Experiment 2, we adjusted the ratio of repeated and novel trials. The experiment consisted of 28 blocks, each containing 32 trials, leading to a total of 896 trials. Unlike Experiment 1, each block included 8 repeated displays and 24 novel displays, maintaining a 1:3 ratio of repeated to novel, as well as predictable to unpredictable trials. This setup aims to mirror the imbalance between predictable cues (valid repeated, 25%) and unpredictable cues (including both invalid repeated and novel trials, in total 75%) in the 50% cue validity condition in Experiment 1. The design of repeated displays followed the classical contextual cueing paradigm, with distractor locations fully predicting the target locations, ensuring 100% cue validity, while in novel displays the randomly presented distractor were not predictive of target location. The other settings of the procedure were same as in Experiment 1.

### 3.2 Results

Data exclusion criteria were same as in Experiment 1. 3.1% of trials were removed.

**Figure 3:**
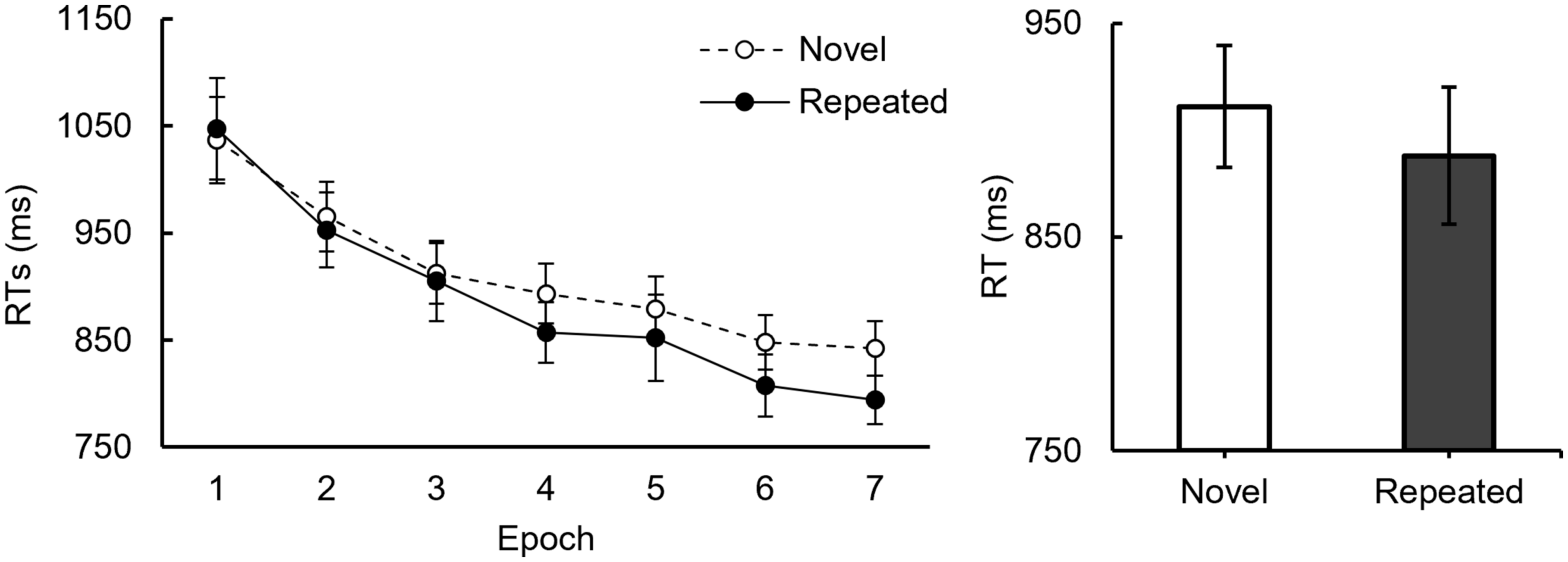
Mean RTs for repeated and novel displays in Experiment 2. Mean RTs as a function of epoch and context condition. The dashed line represents the novel display, whereas the solid line represents the repeated display. The mean RTs for novel and repeated displays are shown on the right side. Error line indicate the standard error.

A 2 (context condition: repeated and novel displays) × 7(epoch) repeated-measures ANOVA was conducted (Figure 3), using Greenhous-Geisser correction due to sphericity violations. Results showed a significant main effect of epoch, *F* (2.19, 50.25) = 42.88, *p* < 0.001, *η_p_^2^* = 0.65, *BF_10_* = 1.30 × 10^26^, and a significant interaction between context condition and epoch, *F* (3.88, 89.25) = 3.16, *p* < 0.05, *η_p_^2^* = 0.12, *BF_10_* = 4.21. The main effect of context condition, however, was not significant, *F* (1, 23) = 3.32, *p* = 0.08, *η_p_^2^* = 0.13, *BF_10_* = 1.10. Further simple effect analyses showed no difference in RTs between repeated and novel displays for epochs 1–6 (*ps* > 0.05), but repeated displays prompted marginally significantly faster RTs than novel displays during epoch 7 (*p* = 0.06), all *p*-values were corrected by Holm-Bonferroni method. These results suggested that the contextual cueing effect was robust despite the 1:3 ratio of repeated to novel displays.

## 4 Experiment 3

Experiments 1 and 2 highlighted the influence of cue validity on the contextual cueing effect. However, it is still unclear whether the absence of contextual cueing effect in low cue validity was due to unsuccessfully contextual learning or inhibited behavioral expression after learning. Previous study indicates that contextual learning (the acquisition of context memory) and expression (the utilization of context memory) are separate processes (Chaumon et al., 2009). Context cues may be implicitly acquired during learning and can manifest as behavioral benefits once attention is directed to them during expression (Jiang and Leung, 2005; Goujon et al., 2009). While Experiment 1 has established the role of cue validity in the contextual cueing effect, it remains unclear whether this influence occurs during the initial acquisition of context cues or during the subsequent manifestation in behavioral search.

To explore this, Experiment 3 divided the experimental procedure into learning and expression phases, inspired by prior research (Brockmole et al., 2006). Participants first underwent a learning phase with 50% cue validity, then to an expression phase with 100% cue validity. If cue validity affects learning, neither phase would show a contextual cueing effect. However, if cue validity only influences the expression phase but not the implicit learning phase, the contextual cueing effect should become apparent in the expression phase once the low cue validity constraint is removed.

### 4.1 Methods

#### 4.1.1 Participants

A total of 19 healthy participants (7 males and 12 females; ages 18–22 years; mean age ± SD = 19.89 ± 1.29 years) were recruited. A G*Power analysis confirmed the sample size was adequate, ensuring a power of 0.80 with 14 participants and 0.95 with 21. All other information regarding the participants is same as in Experiment 1.

#### 4.1.2 Apparatus and materials

Same as Experiment 1.

#### 4.1.3 Design and procedure

**Figure 4:**
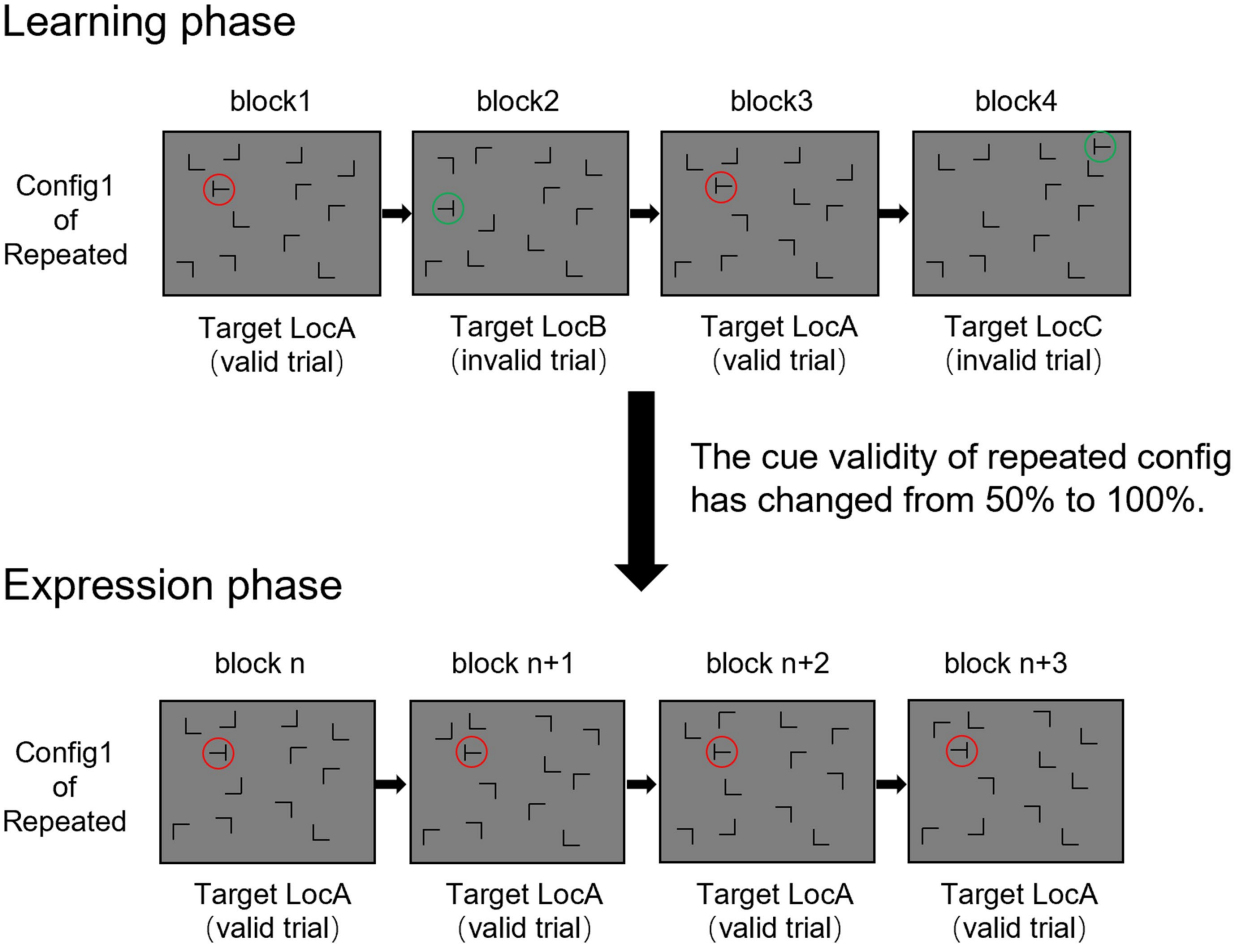
Schematic illustration of Experiment 3. Experiment 3 consists of two phases. The first is learning phase, which includes 4 epochs. Each epoch consists of 4 blocks, with each block containing 32 trials. Half of the trials are repeated displays and the other half are novel displays. Similar to the 50% cue validity condition in Experiment 1, in a typical epoch, repeated displays predict the target location in 50% of the blocks, while the remaining 50% of the target locations are random. In novel displays, distractor locations are presented randomly. Beginning with the fifth epoch, without noticeable transition, cue validity is increased to 100%. The expression phase also includes 4 blocks, each with 32 trials, half being repeated displays and half novel displays. This phase mirrors the 100% cue validity condition from Experiment 1, where the combination of target and distractors in repeated displays consistently predicts the target’s location.

The experiment comprised a learning phase and an expression phase, each comprised four epochs, with four blocks per epoch. Each block contained 32 trials, leading to a total of 1,024 trials. During the learning phase, the design mirrored the 50% cue validity condition from Experiment 1 (Figure 4). Distractor locations were fixed in repeated displays, with a 50% probability of matching a fixed target location and a 50% probability of matching a random target location. In novel displays, distractor locations varied randomly in each trial. The expression phase immediately followed the learning phase, with no noticeable transition. This phase mirrored the 100% cue validity condition (Figure 4), where repeated displays consistently matched fixed targets from the learning phase. The procedure of this phase was identical to that of Experiment 1.

### 4.2 Results

Data exclusion criteria were same as in experiment 1. 3.1% of trials were removed.

**Figure 5:**
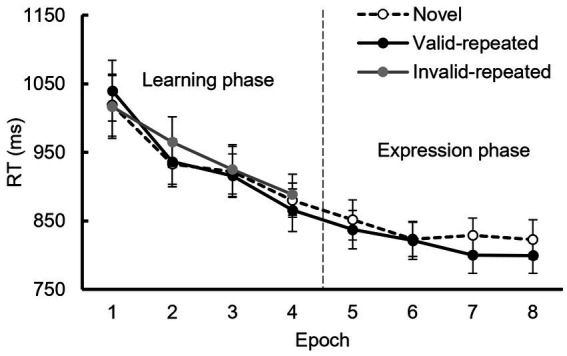
Mean RTs in Experiment 3 for each context condition in the learning and expression phases. Error lines indicate the standard error.

During the learning phase, a 3 (context condition: valid display, invalid display, and novel display) × 4 (epoch: 1–4) repeated-measures ANOVA was conducted (Figure 5), using Greenhous-Geisser correction due to sphericity violations. The results showed a significant main effect of epoch, *F* (1.73, 31.05) = 29.06, *p* < 0.001, *η_p_^2^* = 0.62, *BF_10_* = 4.64 × 10^8^, but no significant main effect of context condition, *F* (1.25, 22.54) = 0.72, *p* = 0.44, *η_p_^2^* = 0.04, *BF_10_* = 0.12, nor a significant interaction, *F* (6, 108) = 1.34, *p* = 0.25, *η_p_^2^* = 0.07, *BF_10_* = 0.21. We extracted the data of the last epoch (epoch 4) in the learning phase. By conducting a paired-samples t-test to compare the differences between valid repeated and novel displays, the results showed that there was no significant difference between them, *t* (18) = 1.17, *p* = 0.26, Cohen’s *d* = 0.27, *BF_10_* = 0.52. Repeated displays did not induce a behavioral advantage over novel displays in the learning phase.

In the expression phase, a 2 (context condition: repeated vs. novel display) × 4 (epoch: 5–8) repeated-measures ANOVA revealed a significant main effect of epoch, *F* (3, 54) = 5.90, *p* < 0.005, *η_p_^2^* = 0.25, *BF_10_* = 16.64. However, neither the main effect of context condition, *F* (1, 18) = 4.16, *p* = 0.06, *η_p_^2^* = 0.19, *BF_10_* = 1.32, nor the interaction, *F* (3, 54) = 1.27, *p* = 0.29, *η_p_^2^* = 0.07, *BF_10_* = 0.34, were significant. The differences between the valid repeated and novel displays were compared in the first epoch (epoch 5) when entering the expression phase. The results indicated that there was no significant difference between them, *t* (18) = 1.17, *p* = 0.12, Cohen’s *d* = 0.37, *BF_10_* = 0.82.Thus, the expression phase showed no significant difference between repeated and novel displays, indicating the absence of the contextual cueing effect.

## 5 Discussion

In this study, we explored how cue validity affects the contextual cueing effect through three experiments. In experiment 1, we manipulated the cue validity of repeated displays in the contextual cueing effect at levels of 100, 75, and 50%. The results showed that high cue validity elicited the contextual cueing effect, whereas low cue validity did not. This indicates that cue validity is a crucial factor in attention allocation and learning, essential for successful application in visual search, as manifested in contextual cueing effect. Experiment 2 supported the finding of Experiment 1 by examining the impact of the ratio between predictable and unpredictable cues on learning. Even when faced with a substantial amount of unpredictable information (75%), participants were still able to learn from the small number of predictable cues. This demonstrates the robustness of the contextual cueing effect and the ability of participants to extract useful information from complex environments. Finally, Experiment 3 differentiated the impact of cue validity on the learning and expression phases, indicating that low cue validity affects cue acquisition during the learning phase rather than the behavioral expression phase.

Visual cues effectively guide attention, enhance visual search, and accelerate learning (Kühl et al., 2012). Cue validity plays a significant role in modulating learning (Griffiths and Thorwart, 2017). When cue validity is high, learning becomes more effective as individuals can rely on reliable information to better construct and test hypotheses (Brehmer, 1980; Wang et al., 2016; Griffiths and Thorwart, 2017). This reliability is particularly beneficial when paired with outcome feedback, improving learning performance (Ruffner and Muchinsky, 1978). Effective cueing reduces cognitive load, which in turn enhances retention and transfer of learning in multimedia environments (Xie et al., 2017). As a result, lower cognitive load due to effective cueing leads to better learning outcomes. Conversely, inconsistent cues can impair the ability to learn and utilize valid cues, diminishing overall learning effectiveness (Wang et al., 2016). The findings from Experiment 1 and 2 of our study align with previous research, further demonstrate the importance of cue validity in the contextual cueing effect. This highlights its impact on the ability of individuals to learn and apply contextual cues for attentional guidance in visual search. High cue validity significantly elicits the contextual cueing effect, while low cue validity did not yield similar results (Figure 2). Cue validity promotes attention allocation based on the proportion of valid trials (Risko and Stolz, 2010). This indicates that attention is preferentially allocated to more predictive cues and away from less predictive ones (Griffiths and Mitchell, 2008; Remillard, 2009; Couperus et al., 2011). In this study, high cue validity supported stable associations between cues and targets, enhancing learning outcomes. Conversely, low cue validity hindered learning by weakening attention allocation.

Learning the configuration of distractors in repeated contexts is a highly efficient and rapid process (Zinchenko et al., 2020). Even when target locations are relocated to fixed positions within repeated contexts, the contextual cueing effect can quickly emerge (Zellin et al., 2014; Zinchenko et al., 2024). At 75% cue validity, participants effectively learned the configurations, as evidenced by poorer performance on invalid repeated trials (Figure 2B). This suggests that participants learned the relationship between distractors and target, thus when target appeared in unpredictable location, attention had to shift from the expected target location to the new one. However, in the 50% cue validity condition, the uncertainty of the cues disrupted the leaning process (Figure 2C). As a result, no contextual cueing effect was observed.

Experiment 2 further supported the findings of Experiment 1, highlighting the critical role of cue validity in the contextual cueing effect. The results indicated that the absence of contextual cueing under low cue validity condition was attributable to the cue validity, rather than the imbalance between predictable and unpredictable trials. Participants gained a behavioral advantage in repeated displays even when predictable trials were less frequent (25%). This suggests that even a lower frequency of predictable trials can yield a substantial contextual cueing effect, provided that cue validity remains a key factor. Meanwhile, it also indicated that even with numerous novel displays, individuals can implicitly extract context cues and achieve significant behavioral facilitation. This result is consistent with findings reported by Yang and Merrill (2015), who observed a similar effect when the ratio of repeated to novel displays was approximately 1:2. However, other studies have shown no contextual cueing effect at 1:4 and 1:5 ratios (Zang et al., 2018; Zinchenko et al., 2018). These differences suggest that the contextual cueing effect is highly sensitive to the proportion of repeated displays. When the proportion of repeated contexts is higher, participants have more opportunities to learn and utilize the configuration of repeated displays, thus enhancing search efficiency. Conversely, as the proportion decreases, opportunities for learning repeated contexts diminish, preventing the formation of a stable contextual cueing effect. Therefore, the proportion of repeated trials is also a key factor affecting the contextual cueing effect.

Current theories on the contextual cueing effect primarily focus on the attentional guidance account and the response facilitation account (Sisk et al., 2019). The attentional guidance account suggests that previous experiences guide attention towards the location that the target is highly likely to appear, speeding up early visual search before the target appears. In contrast, the response facilitation account proposes that prior experiences aid target verification and response selection after the target appears. These findings support the attentional guidance account. Cue validity can influence behavioral performance by modulating attention allocation (Vossel et al., 2006; Swansburg and Neyedli, 2019). In the high cue validity condition, attention was shifted to the target location before the target’s appearance, resulting in a behavioral advantage in a visual search task. Conversely, in the low cue validity condition, fewer attentional resource was allocated to target locations, eliminating the behavioral advantage and eventually the contextual cueing effect. Experiment 3 further emphasized the importance of attention in contextual cueing effect by showing that initial cue acquisition efficiency is crucial to the contextual learning effect. If cues are not effectively acquired during the learning phase, no behavioral advantage can be induced by the cues in the expression phase. This suggests that the absence of the contextual cueing effect in the low cue validity condition does not arise from inhibited expression of learned experience, but from limited attention allocation and unsuccessful implicit acquisition of cue-distractors patterns.

Notably, this study presents findings that differ from recent research, which reported enhanced behavioral performance due to configurations of distractors in repeated displays, even when these configurations cannot predict target locations at all (Chun and Jiang, 1998; Vadillo et al., 2021; Chen et al., 2024). However, the results from Experiment 1 indicate that low cue validity (50%) affects implicit learning and hinders the efficiency of visual research (Figure 2). Chen et al. (2024) introduced the concept of contextual suppression, suggesting that when cues completely fail to predict target locations, the brain adopts more efficient visual search strategies by deprioritizing distractor locations, creating a significant behavioral advantage when searching the remaining locations. The lack of advantage with 50% cue validity may be due to the uncertainty of the cues, making it difficult for individuals to develop a consistent search strategy. Existing research has shown that different levels of cue validity can influence processing strategies (Yang et al., 2019). Therefore, in contextual cueing learning, the brain can also adjust search strategies based on changes in cue validity to achieve optimal behavioral performance.

Across three experiments, cue validity plays a crucial role in generating the contextual cueing effect. High cue validity promoted the learning of links between context cues and target, whereas low cue validity disrupted this learning, especially when cues were coupled with random targets. This disruption might be due to reduced attention allocation, leading to unsuccessful contextual learning. This suggests that observers can effectively utilize reliable cues to form memory associations about the visual displays, leading to the manifestation of the contextual cueing effect. When combined with the results of Zinchenko et al. (2018), a more comprehensive picture emerges. Their study also touched on the influence of reliability factors on contextual cueing. In both our research and theirs, it becomes evident that the reliability, which includes factors such as the general stability and predictability of the visual context, plays a crucial role. If the environment is highly volatile or unpredictable, the contextual cueing effect may be weakened or even absent. This collective evidence indicates that a high reliability of cues is a prerequisite for effective visual scene learning. In order for observers to successfully build up memory associations related to the visual displays through contextual cueing, they need to rely on cues that are consistent, predictable, and relevant.

This study enhances our understanding of the relationship between cue validity and the contextual cueing effect, particularly across different phases. The findings of this study might be informative for the design of more effective learning and training programs, especially in situations where quick extraction of information from complex backgrounds is necessary. The limitations of this study primarily relate to the manipulation of target locations in the invalid repeated condition. Firstly, unpredictable targets were randomly presented across 48 unused locations without strict control over their distribution between inner and outer positions. RTs may be influenced by participants’ search strategies, so future research will investigate these strategies when cues are invalid within the contextual cueing effect. Secondly, to prevent potential target learning effect, future studies will ensure targets in invalid repeated displays are matched to fixed positions rather than being randomly selected. Additionally, future research could explore additional factors that influence the contextual cueing effect, such as individual differences and contextual complexity, or examine the generalizability of these effects in different environments.

## 6 Conclusion

This study examined the impact of cue validity on the contextual cueing effect and its mechanisms through three experiments. We observed that (1) individuals selectively learn context cues based on cue validity, with a complete effect at high cue validity levels (100 and 75%), while low cue validity (50%) impedes the effect; (2) In low cue validity conditions, repeated displays are perceived as invalid cues, negating the effect; (3) Low cue validity disrupts the learning process of context cues rather than its expression in behavioral search.

## Data Availability

The original contributions presented in the study are publicly available. The data can be found here: https://osf.io/f6zxt/.
